# A Study on the Contact Characteristics of Tires–Roads Based on Pressure-Sensitive Film Technology

**DOI:** 10.3390/ma16186323

**Published:** 2023-09-21

**Authors:** Bo Chen, Pengbo Ding, Guojie Wei, Chunlong Xiong, Fangli Wang, Jinfeng Yu, Huayang Yu, Yuxun Zou

**Affiliations:** 1 School of Transportation and Civil Engineering and Architecture, Foshan University, Foshan 528000, China; 2 Guangdong Expressway Co., Ltd., Guangzhou 510623, China; pengboding@163.com; 3 Guangdong Provincial Government Loan Repayment Highway Management Centre, Guangzhou 510199, China; 13926637176@163.com; 4 Guangdong Nan Yue Transportation Lian-Ying Expressway Management Office, Guangzhou 510199, China; 5 School of Civil Engineering and Transportation, South China University of Technology, Guangzhou 510006, China; cthgclx@mail.scut.edu.cn (C.X.); 202221010188@mail.scut.edu.cn (J.Y.); huayangyu@scut.edu.cn (H.Y.); 6 China Road and Bridge Co., Ltd., Beijing 100011, China; wangfl@crba.com

**Keywords:** tire–road contact, pressure-sensitive film, asphalt pavement, texture depth, contact reduction rate, peak contact stress

## Abstract

Tire–road characteristics are a critical focus of research in the automotive and transportation industries. On the one hand, the research can help optimize tires’ structural design; on the other hand, it can analyze the mechanical response of the pavement structure under the vehicle load. In addition, the non-uniformity distribution of the tire ground stress will also have a direct impact on the skid resistance, which determines the driving safety. Due to the limitation of testing technology, the measurement of tire ground pressure was mainly carried out on a flat test platform, ignoring the roughness of the actual pavement surface texture. The tire–road contact characteristics research on the macro-texture and micro-texture of asphalt pavement needs to be broken through. A high-precision pressure-sensitive film measurement system is utilized to examine the actual contact characteristics between two types of automobile tires and three types of asphalt pavement in this paper. The influence law of pavement texture and patterned tires on the contact area and stress was explored, and the concentration effect of tire–road contact stress was evaluated. The results indicate that the contact area of grounding tires exhibits a nearly linear relationship with tire inflation pressure and load. Notably, the change in load has a more significant influence on the contact area than tire inflation pressure. On asphalt pavement, the contact reduction rate decreases by approximately 5–10% for block pattern tires and 10–15% for longitudinal pattern tires. Furthermore, as the texture depth of the pavement increases, the contact area between tires and the pavement texture decreases. The actual tire–road interface experiences significant stress concentration due to the embedding and meshing effects between the tire and road surface. Even on a flat steel surface, the peak stress at the edge of the tread block exceeds the 0.7 MPa design load, which is about 2.5–3 times higher than the design uniform load. The peak stress between the tire and asphalt pavement reaches 4–10 times the design uniform load, with a rising trend as the pavement texture depth increases. This study can provide relevant experimental technical support for tire design and functional design of asphalt pavement.

## 1. Introduction

Tire contact problems have traditionally been the focus of research in the automotive tire industry and the transportation industry. On the one hand, the study of tire contact characteristics can help optimize the tire structure design and improve the comprehensive grounding performance of automobile tires [1,2,3]; on the other hand, the tire contact characteristics can analyze the mechanical response of the pavement structure under the vehicle load, which can help one to design the appropriate pavement structure combinations and thicknesses [4,5,6]. In addition, the non-uniform distribution of the tire grounding stress will also have a direct effect on the skid resistance of the road surface, which affects the driving stability and safety of vehicles [7,8]. Tire rubber is a polymer viscoelastic material. Different rubber materials have different composite moduli, but compared with the road material, their deformation is larger. The deformation of tire rubber is also related to temperature; when the rubber temperature is less than the glass state temperature, the rubber strain is small, and at high temperatures (when the temperature is higher than the critical temperature of viscous flow), the rubber shows a viscous flow state [9,10]. The normal use temperature range of automobile tires is in the high elastic state of rubber, which can produce large deformations and recovery after the force, resulting in a more complex tire–road contact state [11,12]. In the tire manufacturing industry, the testing and simulation analysis of different tires under various working conditions often involves replacing smooth surfaces with rough pavements. However, this approach does not accurately reflect the actual tire–road contact characteristics. When designing pavement structures, the tire–road contact pressure is typically assumed to be a uniformly distributed circular or equivalent rectangular load. This assumption is widely used due to its simplicity in illustration and mathematical calculations [13]. This approximate hypothesis has minimal impact on design results and analysis for thick pavement structures. However, the structural response of thin asphalt pavements is highly sensitive to the shape and distribution of tire–road contact pressure. Studies have shown that predicting the fatigue life of asphalt pavements under measured non-uniform loads yields better agreement than traditional uniform loading assumptions [14]. Furthermore, tire–road contact behavior is closely associated with road travel safety. The friction between the road surface and tires arises from the hysteresis force generated by the Macro-texture of the road surface and the adhesion force generated by the micro-texture. Both of these forces originate from the tire–road contact stresses [15,16]. Therefore, road skid resistance is essentially a contact mechanics problem. Excellent vehicle braking performance is contingent upon the tire–road contact interface characteristics.

In the field of tire–road contact mechanical properties, early studies relied on empirical models or analytical methods based on tests, leading to the development of various simplified theoretical models such as the Hertz contact model, the G–W (Greenwood–Williamson) contact model, and the W–A (Whitehouse–Archard) contact model [9,17,18,19,20,21]. Persson proposed a tire–road contact model based on the roughness power spectral density function, which reduces the tire–road contact problem to the elastic contact behavior between a smooth rubber plane and a rough surface with fractal features. This model describes the incomplete contact state between the road surface and rubber and theoretically explores the contact state between the rough road surface and the tire [22,23]. Due to the nonlinearity of tire materials, large deformations, and complex pavement roughness involved in tire–road interactions, researchers both domestically and internationally have turned to finite element software to simulate tire–pavement contact interactions. Al-Qadi et al. conducted finite element simulations based on experimental data of tire grounding stress. They built a three-dimensional inflatable tire model to simulate the interaction between the tire and rigid pavement and predicted pavement damage using the traditional dual-tire structure, new and old wide-face tires, and other configurations. Their findings highlighted the sensitivity of thin-layer asphalt pavement structures to the non-uniform distribution of vertical contact stresses, emphasizing the importance of considering this in empirical mechanics design [24,25]. Scarpas et al. developed a dynamic contact model considering pavement texture patterns and the deformation of pneumatic tires. They simulated and analyzed the effects of energy dissipation and temperature changes on tire/road friction during tire rolling, braking, and cornering [26]. In recent years, some scholars have explored the contact effects between tire tread compounds and rough substrates through finite element models. They attempted to propose simulation methods for calculating the sliding friction coefficient, achieving certain success. However, further investigation is needed to determine the scope of application for these methods [27,28]. Overall, the studies above had certain limitations. They did not fully consider the fine-scale rich texture and macro-scale rough structure of asphalt pavement surfaces and their multi-scale cross-section contact characteristics. Additionally, due to technical constraints, the distribution law and the degree of non-uniformity of contact pressure between the tire and the actual pavement could not be evaluated effectively and quantitatively. Undoubtedly, tire–road contact stress testing technology is crucial for studying tire–road contact characteristics, and finding appropriate methods for testing and evaluating tire–road contact characteristics holds significant research and application value for both the tire industry and the road industry.

Tire–road contact pressure distribution testing methods include pressure cell, triaxial load pin, piezoelectric sensor, and pressure-sensitive film [29,30,31]. The accuracy of tire grounding characteristic measurements is affected by the sensors’ size, spacing, and sensitivity between the tire and the ground. Pressure cell and triaxial load pin test methods are relatively simple to operate, but have lower overall measurement accuracy [32]. In 1988, the Tekscan Company in the USA developed the T-Scan system to measure the bite force of dental patients [33]. The Tekscan pressure sensor consists of two polyester films (approximately 0.2 mm thick) with longitudinal and transverse pressure-sensitive semiconductor material forming a pressure sensing point array. When tire pressure is applied to the sensing points, the semiconductor resistance changes accordingly, providing measurements of the tire–ground pressure distribution. The system has a resolution of 3.048 mm × 3.048 mm and can test the dynamic pressure distribution of tires at speeds of up to 30 km/h. Liang et al. utilized the Tekscan pressure measurement system to assess the grounding performance of radial tires and described changes in the geometric characteristics of the tires’ contact imprint profile on a steel plate [34]. However, tire grounding pressure measurement is typically conducted on a flat test platform, overlooking the roughness of actual road surfaces, and there is still a need to make breakthroughs in studying tire–road contact characteristics on macro- and micro-pavement textures. With the development of various new materials, some high-precision materials have achieved better application results [35,36]. In the early 1980s, a pressure-sensitive film system called Prescale, produced by the FUJI Company in Japan, was introduced. This system utilizes a chemical reaction to measure grounding pressure and has been applied in various fields such as automotive components, LCD polarizers, semiconductor processing, printed circuit boards, and pressure distribution measurement. Tielking et al. used pressure-sensitive film to measure tire–road pressure and discovered significant non-uniformity in the tire–road pressure, with vertical stress at certain locations exceeding the range of the pressure-sensitive film [37]. With advancements in technology, the measurement system has become highly accurate and capable of recognizing contact stress units more minor than the 0.5 mm scale, making it a promising tool for research applications [38,39].

This study primarily employs a high-precision pressure-sensitive film measurement system to examine the actual contact characteristics between various automobile tires and different types of asphalt pavements. The aim is to investigate how pavement texture influences the contact area and contact pressure of treaded tires and evaluate the concentration effect of tire–road contact stresses. The findings of this research can promote the understanding of tire–road contact characteristics considering tire pattern and pavement texture characteristics and provide valuable technical support for tire design and the functional design of asphalt pavements.

## 2. Tire–Road Contact Mechanism

**Figure 1:**
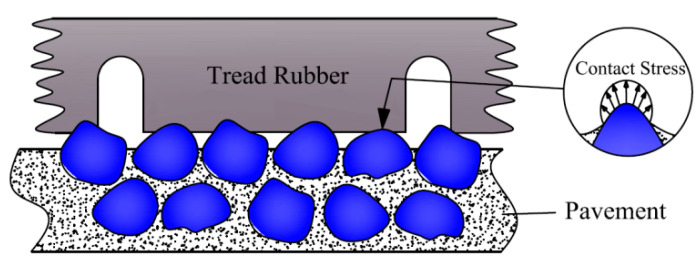
Schematic of tire–road contact.

The World Road Association (PIARC) classifies road surface characteristics into four categories: microtexture, macrotexture, megatexture, and roughness [40]. When a rubber tire comes into contact with an uneven road surface texture, it undergoes significant deformation at the convex parts of the road surface while making actual contact with the texture peaks, as depicted in Figure 1. This is because the rubber hardness is much lower than that of the road surface aggregates, and the tire has a non-solid inflatable structure. Asphalt pavement forms a macro-contour with raised aggregate particles on the tread rubber, resulting in localized deformation and embedding. On asphalt pavements, the protruding aggregate particles that constitute the macroscopic contour result in localized encasement and embedding deformation of the tire rubber. The quality and processing techniques of the aggregate particles lead to differences in their shapes, while the gradation design and construction processes affect the interlocking state between coarse particles. This results in varying upright states of the road surface particles (forming right angles, acute angles, obtuse angles, etc.) and texture forms, ultimately influencing the distribution of contact stresses on the tires.

In 1881, Heinrich Rudolf Hertz proposed a theoretical model of elastomeric contact based on his observations of the elastic deformation properties of glass lenses interacting with each other [19]. According to the classical Hertz contact theory, the contact stresses between two curved surfaces are determined by external loads, the radii of curvature of the two contacting surfaces, and the elastic modulus. The contact stress and contact radius can be calculated using the following equations:

For double orb contact:

(1) $$p(r)=\frac{3P}{2\pi a^{2}}\sqrt{1-{\left(\frac{r}{a}\right)}^{2}}$$

(2) $$a={\left(\frac{3PR}{4E^{*}}\right)}^{1/3}$$

For double cylinder contact:

(3) $$p(x)=\frac{2P}{\pi bL}\sqrt{1-{\left(\frac{x}{b}\right)}^{2}}$$

(4) $$b={\left(\frac{2PR}{\pi LE^{*}}\right)}^{1/2}$$

where *R* is the relative radius of the contact surface, $\frac{1}{R}=\frac{1}{R_{1}}+\frac{1}{R_{2}}$; $E^{*}$ is the contact modulus, $\frac{1}{E^{*}}=\frac{1-\nu^{2}}{E_{1}}+\frac{1-\nu^{2}}{E_{2}}$; *P* is the applied load; *a* is the contact radius of the two spheres; *b* is the contact width of the two cylinders; p(r) is the stress at radius *r*; and p(x) is the stress at *x* from the center.

However, there are apparent differences between Hertz contact theory and tire–road contact: (1) the tire structure is a combined structure with pneumatic pressure and large contact deformation; (2) the tread rubber of the tire has transverse and longitudinal patterns, the hardness of each part is not uniform, and the actual road surface is not a smooth contact plane. Therefore, it is difficult to obtain an accurate stress distribution using the classical Hertz theory.

**Figure 2:**
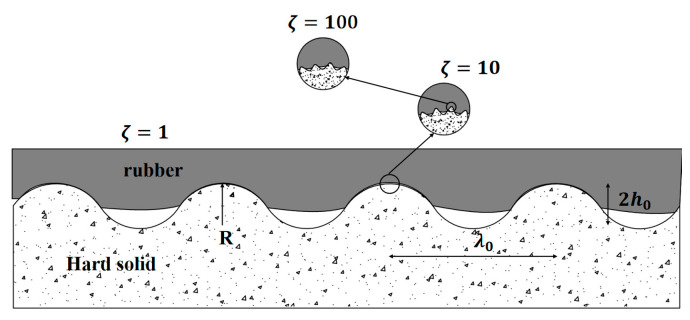
Actual contact state between pneumatic tires and rough road surfaces.

Subsequently, Persson simplified the tire–road contact problem into the behavior of elastic contact between a smooth rubber plane and a rough surface with fractal features and proposed that different contact states of rubber and a hard road surface can be observed by using different amplification factors, and that the effective contact area is linked to the amplification factor [22], as shown in Figure 2.

Persson’s theory of contact calculation is as follows:

(5) $$P(\zeta )=\frac{A(\zeta )}{A_{0}}=\frac{2}{\pi }\int_{0}^{\infty }dx\frac{\sin x}{x}\exp[-x^{2}\int_{1}^{\zeta }d\zeta^{\prime }g\left(\zeta^{\prime }\right)]x$$

among which

(6) $$g(\zeta )=\frac{1}{8}q_{L}q^{3}C(q){\int d\phi \left|\frac{E(qv\cos\phi }{(1-v^{2})\sigma_{0}}\right|}^{2}$$

In the above equation, ζ is a scaling factor; A(ζ) is the actual contact area of the pavement with the wavelength of $\lambda =\frac{L}{\zeta }$; L is the lateral size of the road surface; λ is the length scale of the road surface; $P(\zeta )=\frac{A(\zeta )}{A_{0}}$ is the macroscopic area ratio coefficient; $A_{0}$ is the nominal contact area when the amplification factor is one; $q_{L}$ is the fundamental frequency; $q=q_{L}\zeta$;C(q) is the power spectral density function of the pavement; *E* is the rubber composite modulus; ***v*** is the slip velocity; ϕ is the directional angle; ν is the Poisson’s ratio of the rubber; and $\sigma_{0}$ is the average contact pressure.

Persson deduced the contact theory that, when the amplification factor, the effective contact area between the tire and the road surface, is in the full contact state with no gaps, gaps between tires and road surfaces can be gradually observed when the amplification factor is greater than one, leading the actual contact area to be smaller than the nominal contact area. As the amplification factor increases, the actual contact area becomes smaller. Compared with Hertz’s contact theory, Persson’s contact theory can better describe the contact between the tire rubber and the actual road surface. However, for the contact stress of the tire, the average contact pressure is still used to characterize this.

## 3. Prescale Pressure-Sensitive Film Testing Techniques

### 3.1. System Description

**Figure 3:**
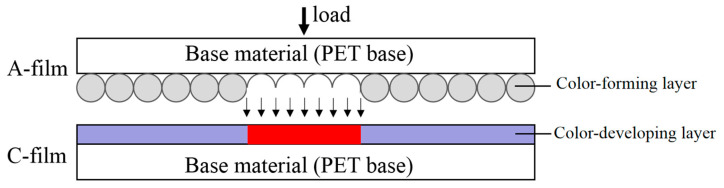
Schematic of two-sheet-type pressure-sensitive film.

Prescale film, manufactured by the FUJI company in Japan, is available in two types: double-slice and single-slice. The double-slice type has one slice uniformly coated with micro capsule materials on top, while the other slice is coated with color-developing materials that chemically react with the reagent released from the ruptured capsules. The color-developing reaction of the film is illustrated in Figure 3. The size and strength of the micro capsule materials are directly related to the magnitude of the contact pressure. Larger micro capsule materials have higher compressive strength compared with smaller ones. Additionally, for micro capsule materials of the same size, the thickness of the cell wall also plays a role. Thinner cell walls are more susceptible to damage under low-pressure conditions, while micro capsule materials with thicker cell walls can withstand larger external loads. The pressure-sensitive film utilizes micro capsule materials that come in dozens of different sizes and cell wall strengths, ranging from a few micrometers to tens of micrometers. By combining different sizes and thicknesses of micro capsule materials, precise measurements of pressure distributions can be achieved. The color density after the reaction is determined by the dosage of the micro capsule material’s color-forming material and the amount of color-developing material involved in the reaction. The pressure-sensitive films have a uniform single-layer thickness of 90 μm and a minimum measurement area of 0.125 mm× 0.125 mm.

Currently, Prescale pressure sensing film is categorized into eight pressure levels based on the effective range of the micro capsule materials, covering a stress test range from 0.05 to 300 MPa. Among these, the super high pressure (HHS), high pressure (HS), and medium pressure (MS) pressure sensing films are the mono-sheet type, while the remaining pressure sensing films are the two-sheet type. Detailed specifications and corresponding range parameters can be found in Table 1. Mono-sheet-type (high pressure) pressure sensing film is usually used in the mechanical industry, while two-sheet-type pressure sensing film is recommended for tire grounding pressure testing.

The pressure-sensitive film test system can be calibrated and converted to pressure values based on the temperature and humidity of the testing environment. Pressure-sensitive film measurements are less susceptible to environmental and human factors. Moreover, placing a double-slice film (0.09 mm × 2) between the tire and the road surface does not significantly alter their contact characteristics. The pressure distribution data at the contact interface are processed, quantized, and stored in a two-dimensional matrix. These data can be further represented as a compact matrix-type discrete function using the following mathematical formula:

(7) $$F=\left[\begin{array}{ccc} f(0,0) & \cdots & f(0,n)\\ \cdots & \cdots & \cdots \\ f(m,0) & \cdots & f(m,n) \end{array}\right]$$

where *F* is the total contact pressure value, N; *f* (*m*, *n*) is the average value of contact stress in the single-point contact area, MPa.

### 3.2. Test Procedure

**Figure 4:**
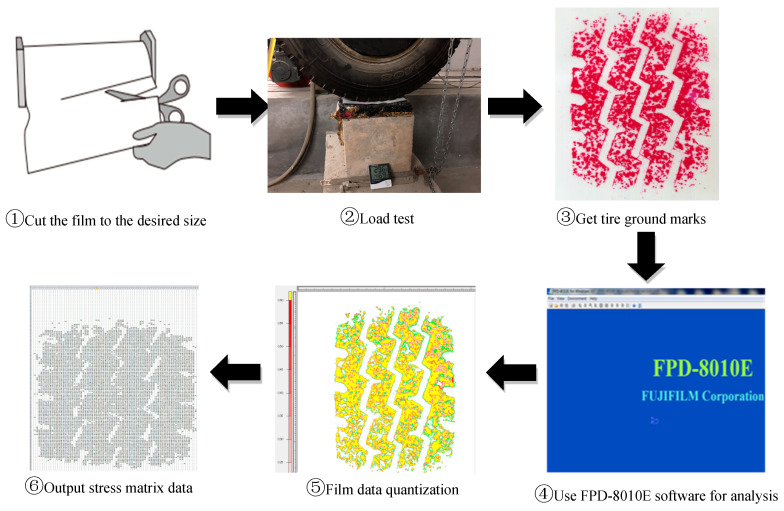
Schematic of pressure-sensitive film operation process.

The pressure-sensitive film testing process is depicted in Figure 4 and is outlined below:

(1) Determine the outer contour size of the effective contact area of the tire based on the tire size with information on the tire pressure and load.
(2) Correctly cut the film to the proper size (usually color-forming A film is in a black bag for a transparent effect, and color-developing C film is in a blue bag for a milky white color).
(3) Place the rough side of each film facing each other, trying to avoid rubbing against each other and touching water to prevent loss of film micro capsule materials, and place the films between the tire and the road surface.
(4) Apply the tire load and maintain static pressure for 2 min or instantaneous pressure for 5 s.
(5) Remove the color-developing C film, leave it for 60 min until the color density is gradually stabilized, and then read the characteristic map of contact stress distribution using a Perfection^TM^ V300 Photo type CCD special scanner (Epson Company, Tokyo, Japan) and a color-correction board with an image resolution of 0.125 (200 dpi). Then, use the supporting FPD-8010E pressure image Digital Measurement and Analysis System V2.0 for numerical analysis.
(6) Perform numerical quantification and statistical analysis of contact stress values of different specification films based on the MATLAB (Version 2016b, and the latest version after that) platform.

### 3.3. System Error Analysis

**Figure 5:**
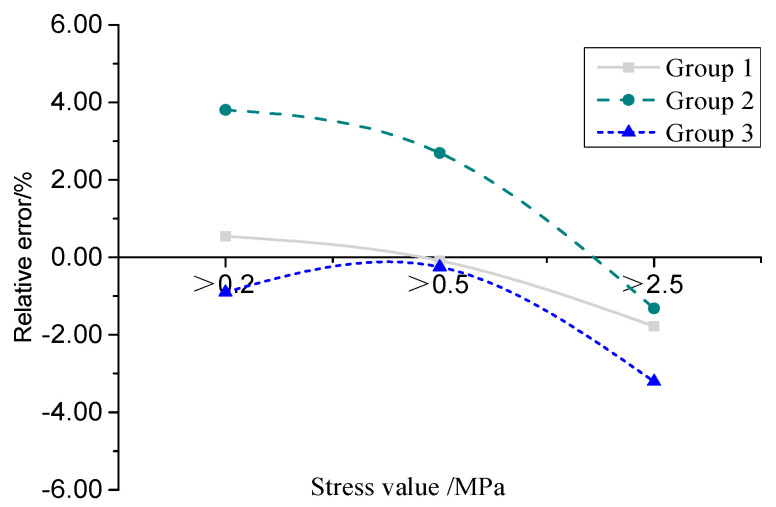
Error analysis of pressure film test system.

In this study, four types of pressure-sensitive films, namely 4LW, LLLW, LLW, and LW, were utilized to test the contact stresses of small automobile tires with a load of 7.04 kN and a tire pressure of 400 kPa. To analyze the measurement error of the test results, tire grounding pressure tests were conducted using three sets of these four types films under a constant single-wheel load. Each film loading test was repeated three times, and the imprint data were collected. The effective contact area within the effective range of each type film was selected as a comparison index. The average value of the effective contact area was calculated for each group of repeated tests, and we separately counted the contact area within the effective range of different film types. Specifically, for the 4LW film, the area of the stress point was greater than 0.2 MPa; for the 3LW film, it was greater than 0.2 MPa and 0.5 MPa; for the 2LW film, it was greater than 0.5 MPa and 2.5 MPa; and for the LW film, it was greater than 2.5 MPa. By comparing and analyzing the contact area of two groups of adjacent specification films and calculating the relative error, as shown in Figure 5, it was observed that the error within the effective range of the pressure-sensitive film measurement data could be controlled within ±4%. This indicates that the measurement error of the pressure-sensitive film measurement system is stable and reliable, meeting the requirements for research and application.

## 4. Materials and Methods

### 4.1. Test Raw Materials

The stress distribution at the tire–road contact interface is closely related to the roughness of the pavement texture, and the variability in the roughness of the pavement texture depends mainly on the gradation design of the asphalt mixture. Four common pavement structures are used in this study: AC-13, SMA-13, and OGFC-13. Shell SBS-modified asphalt (PG76-22) is used for AC and SMA mixtures, and high-viscosity modified asphalt is used for OGFC; the test results are shown in Table 2. The test method mainly refers to Standard Test Methods of Bitumen and Bituminous Mixtures for Highway Engineering (JTG E20-2011, China [41]). Coarse aggregate is pyroxene crushed rock, and the main performance indexes are shown in Table 3, while the fine aggregate (0–3 mm) is limestone mechanized sand, and the main performance indexes are shown in Table 4. The test method mainly refers to Test Methods of Aggregate for Highway Engineering (JTG E42-2005, China [42]).

The gradation design of the mixture is shown in Table 5. According to the above gradation composition, the Marshall test determined the optimal oil–rock ratio, and the dosages of mineral powder in three different asphalt mixtures, AC-13, SMA-13, and OGFC-13 specimens, were 4%, 10%, and 4%, respectively; the optimal oil–rock ratios were 5.1%, 6.2%, and 5.0%, in that order. In addition, 0.35% lignin fiber was added to SMA-13 to ensure its structural stability. The rutted plate (300 mm × 300 mm × 50 mm) was molded under the optimal asphalt–aggregate ratio. The test method mainly refers to Standard Test Methods of Bitumen and Bituminous Mixtures for Highway Engineering (JTG E20-2011, China).

### 4.2. Test Tires and Working Conditions

**Figure 6:**
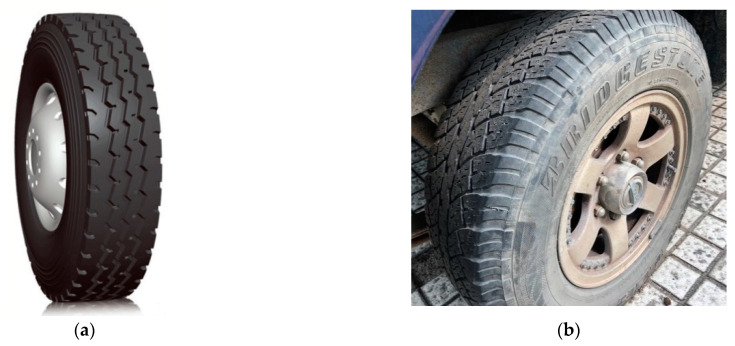
Test tire: (**a**) truck tires; (**b**) car tires.

(1) The test uses a more representative light truck towards the tread all-wire radial tires: tire specifications 7.00R16LT, rim specifications 5.5 F, 12-ply grade, single-wheel rated load of 12.15 kN, the standard tire inflation pressure is 670 kPa, as shown in Figure 6a.
(2) A car block tread radial tire was chosen as a comparison in order to analyze the differences between the contact characteristics of different tires. Specific parameters: tire size 215/75 R15, single-wheel rated load 8 kN, standard tire inflation pressure 300 kPa, as shown in Figure 6b.

### 4.3. Loading Test Platform

**Figure 7:**
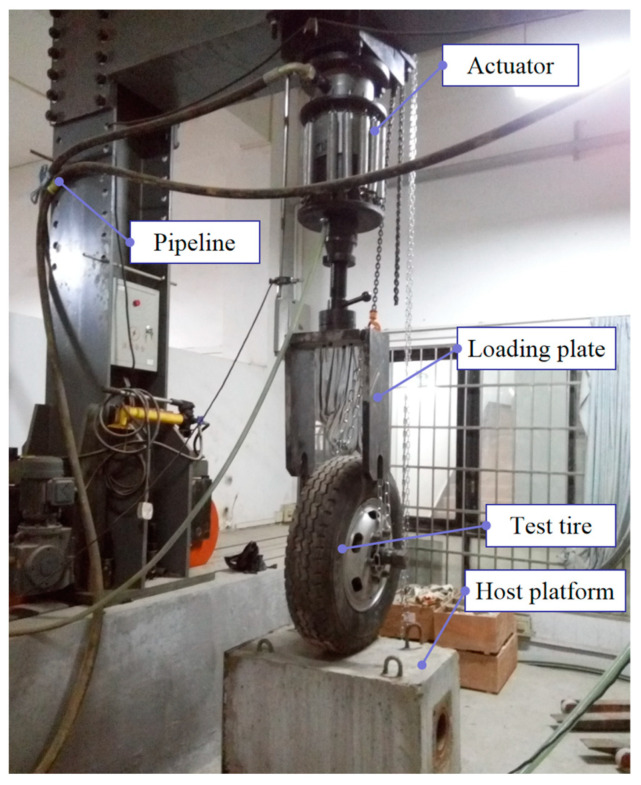
Tire loading test device.

The PMW500 electro-hydraulic fatigue pulsation test system was modified so that the actuator could apply dynamic/static loads to the test tire. The loading system mainly consists of pulsator unit, control system, actuator, pipeline, test protection device, test tire, loading plate, and host platform. It can simulate the loading effect of tires on road surfaces under parking or driving conditions, as shown in Figure 7.

### 4.4. Test Process

**Figure 8:**
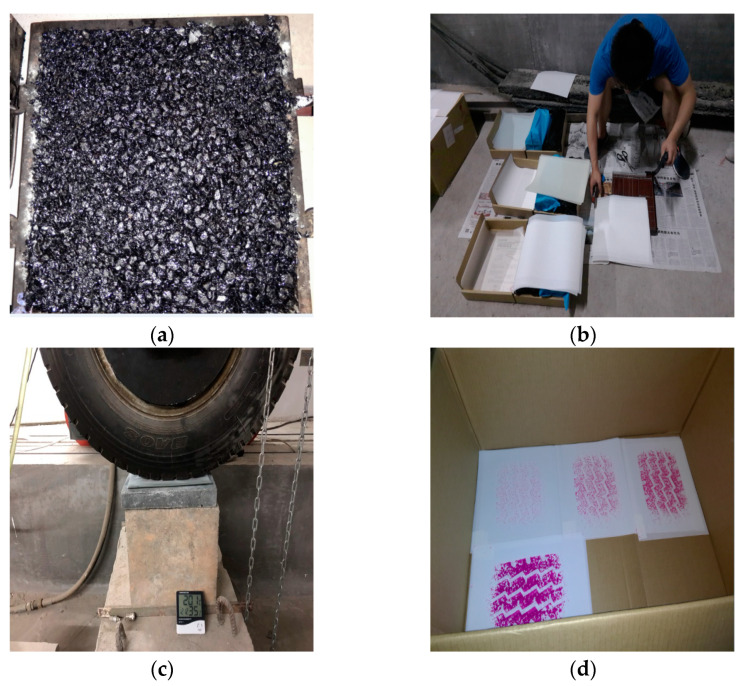
Pressure-sensitive film operation flow: (**a**) rutting plate molding; (**b**) film cutting; (**c**) load test; (**d**) obtaining film imprints.

(1) Mold different asphalt-mixture rutted slabs and measure the tectonic depth (TD) of each asphalt-mixture rutted slab specimen using the sand spreading method.
(2) According to the pre-test attempts, for car tires mainly use 4LW, LLLW, and LLW film types to obtain the tire and road contact stress distribution; for truck tires, due to the hard rubber of the tread and the increase in the single-wheel load, it is necessary to use 4LW, LLLW, LLW, and LLW film types to obtain the complete stress distribution information.
(3) Different types of film are placed between the tire and the road surface. In order to minimize the stress diffusion effect of the film, only one type (two pieces) of test film is used at a time. The PMW-500 is used to apply different static loads to the test tires for a continuous loading time of 2 min or more to ensure the adequate chemical reaction of the micro capsule materials.
(4) Record the temperature and humidity of the test site using a thermo-hygrometer, and determine the corrected model of pressure and color density through the calibration function of the system.
(5) After the reaction of color-developing reagent is fully stabilized, the test films are calibrated and scanned by the software platform of FPD-8010E. Finally, the test results of different types of film are numerically quantified and statistically analyzed by the MATLAB software. The main test process is shown in Figure 8.

## 5. Analysis and Discussion of Results

### 5.1. Analysis of the Tires’ Grounding Imprints

Taking truck all-wire radial tires as an example, the contact imprints between tires and flat steel plates were obtained under different tire pressures and loads to study the effects of tire pressure and load on the grounding imprints of tires.

**Figure 9:**
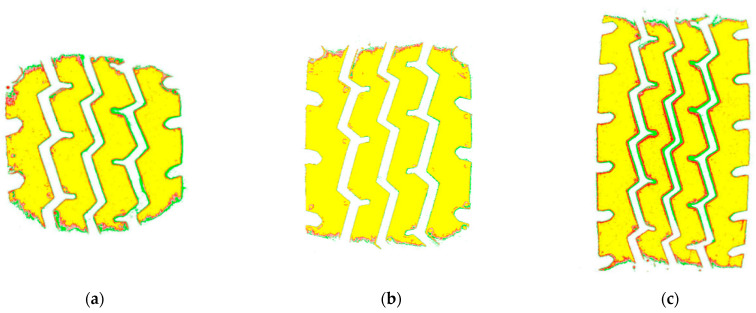
Grounding imprints under different loads: (**a**) 9.5 kN; (**b**) 12.15 kN; (**c**) 25 kN.

**Figure 10:**
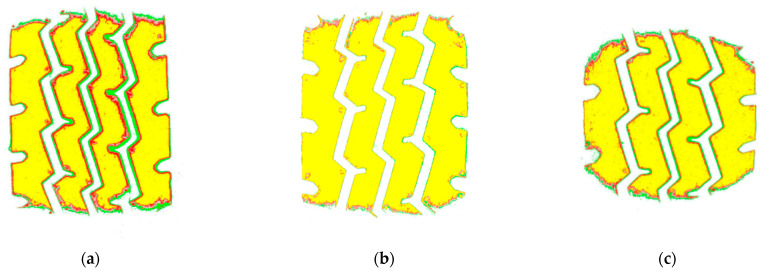
Grounding imprints at different air pressures: (**a**) 400 kPa; (**b**) 670 kPa; (**c**) 900 kPa.

Figure 9 shows the imprints of the light truck tire at 670 kPa tire pressure and different loads. It can be seen that the grounding imprint of this type of tire at 12.15 kN (rated load) is nearly rectangular. When the load is less than the rated load, i.e., 9.5 kN (about 0.78 times the rated load), the tire imprint is nearly circular. When the load is more than the rated load, i.e., 25 kN (about two times the rated load), the tire imprint in the direction of the width of the tire remains unchanged, and the length of the tire imprint increases. The shape of the imprint remains approximately rectangular. The grounding imprints of truck tires at three different air pressures and rated loads (12.15 kN) are shown in Figure 10. As the air pressure decreases, the width of the tire grounding imprint remains unchanged, while the length increases, and the shape of the imprint is approximately rectangular, which is close to the contour at standard tire inflation pressure (670 kPa). As the tire inflation pressure increases, the length of the tire imprint decreases, and the shape of the imprint tends to be round.

**Figure 11:**
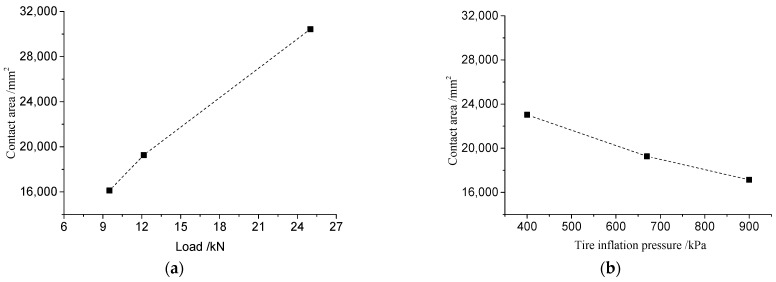
Changing the law of tire grounding area: (**a**) different tire load; (**b**) different tire inflation pressure.

As can be seen from Figure 11, with the increase in load, the tire grounding area increases, and the area and the increase in load presents a nearly linear relationship, with a correlation coefficient of 0.9975. With the increase in tire pressure, the tire contact area decreases, and the area and the change in tire pressure also present a nearly linear relationship, with a correlation coefficient of 0.9872. Based on the slopes of the change in contact area with tire pressure and load (see Table 6), it can be seen that, within the range of tire operating condition changes, the magnitude of the effect of the change in load on the contact area is greater than the effect of tire pressure.

### 5.2. Analysis of the Tire Contact Reduction Rate

**Figure 12:**
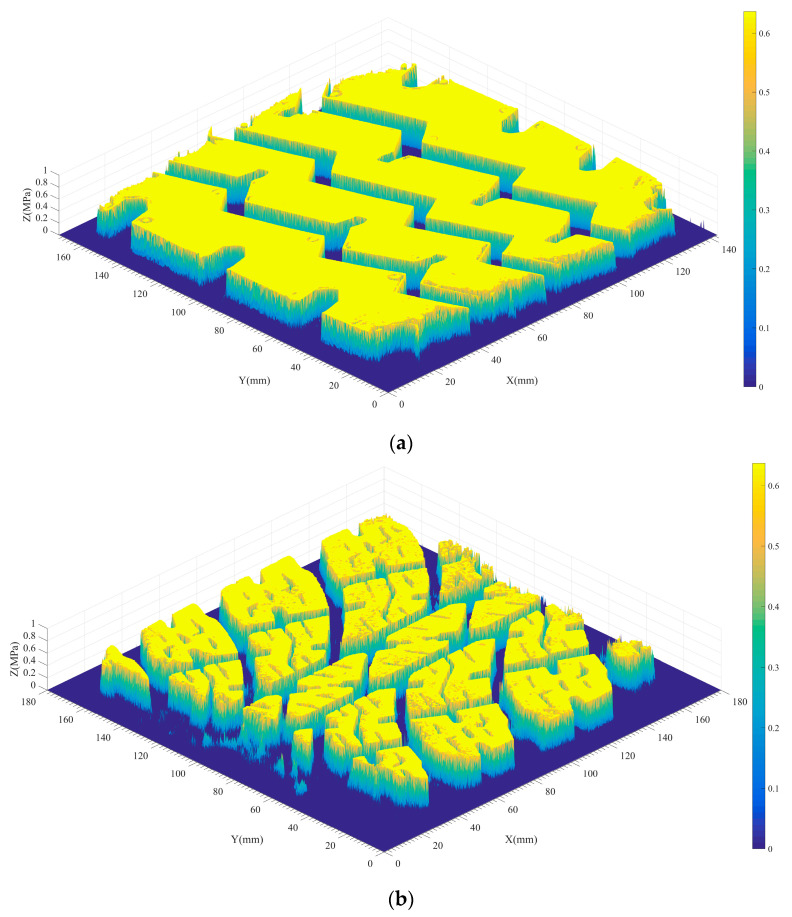
Contact stress distribution of tires with different tread patterns: (**a**) longitudinal tread tires; (**b**) block tread tires.

The effective contact characteristics and stress distribution of tire treads were analyzed using truck longitudinal tread tires and car block tread tires as test tires, as shown in Figure 12.

**Figure 13:**
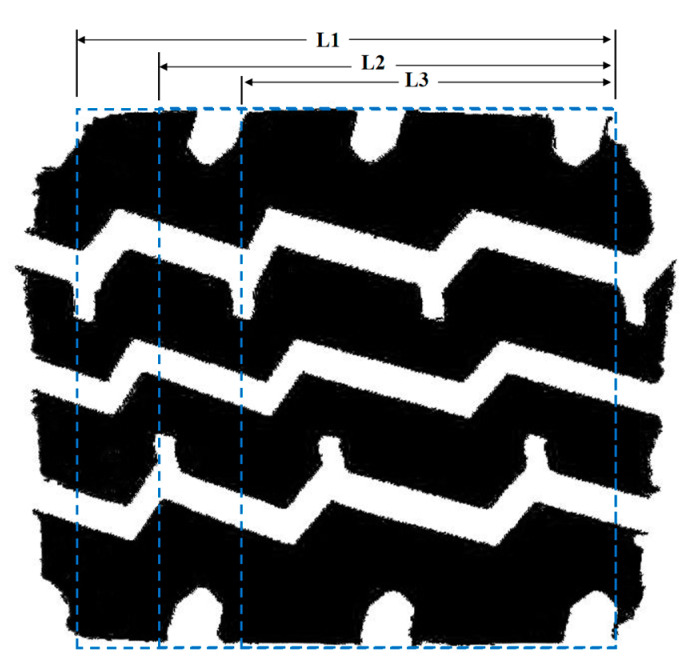
Schematic diagram of tire tread reduction rate calculation (Note: L1, L2, and L3 represent different length values).

The measurement of the tire tread reduction rate is commonly represented by the ratio of the actual grounding area of the tire to the contour area of the imprint. However, to accurately assess the tire grounding imprint under different operating conditions, it is necessary to define the contour conditions. Based on the previous analysis, it was determined that the imprint contour of the tire is approximately rectangular when the tire operates under its rated load and pressure. The variations in tire pressure and load primarily impact the length of the imprint. With this in mind, the length (L) of the intercepted imprint was adjusted within the area of the imprint. Each imprint was then tested five times to calculate the ratio of the effective contact area to the area of the imprint. This process is schematically illustrated in Figure 13.

**Figure 14:**
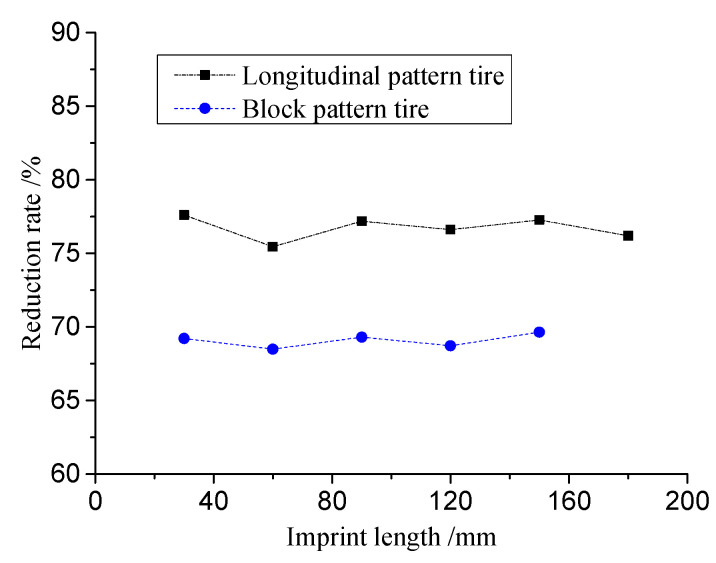
Tire tread reduction ratio.

The contact area ratio was calculated with different imprint lengths at 30 mm intervals as the tire contact reduction rate. The results are presented in Figure 14. For truck longitudinal pattern tires, the reduction rate ranges from 75% to 80%, while for car block pattern tires, the reduction rate is approximately 70%. Calculating the average values of the test data, the average reduction rate for longitudinal pattern tires is determined to be 76.72%, while the average reduction rate for block pattern tires is 69.08%. These findings align with mainstream tire research in the market, which suggests that longitudinal tread tires have an effective contact reduction rate of 75% to 80%, cross-groove tires have a rate of 70% to 75%, and off-road tires have a rate of 60% to 65%. In this study, the reduction rate results for the longitudinal pattern tire are consistent with previous research, while the reduction rate for the block pattern tire falls within the range observed for off-road and cross-groove tires.

### 5.3. Analysis of Contact Reduction Rate on Different Pavements

**Figure 15:**
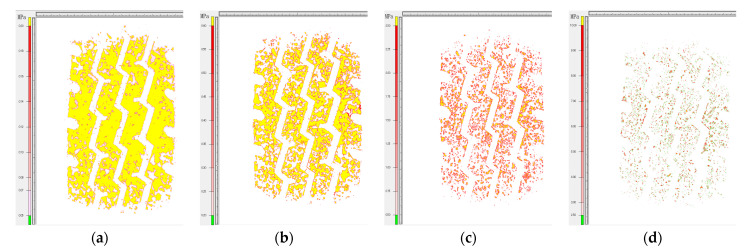
Grounding imprints of truck tires on asphalt pavement (AC-13, for example) under rated conditions: (**a**) 4LW; (**b**) 3LW; (**c**) 2LW; (**d**) LW.

Tire rubber is not a completely flexible structure, and the actual road surface is not a flat plane, so the actual contact between the tire and the asphalt pavement is not a complete full contact state, but a partial contact state. The partial contact of the asphalt pavement texture further leads to a decrease in the reduction rate of the tire–road contact. In order to obtain the effective contact of the tire under the actual pavement texture, the contact area of different tires with different asphalt pavements was measured under different working conditions (load and tire pressure), as shown in Figure 15 below.

**Figure 16:**
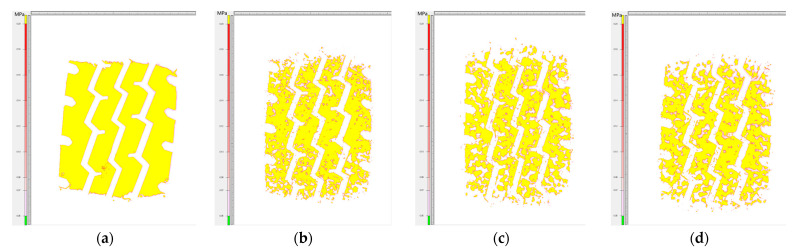
Tire–road contact condition: (**a**) steel plate; (**b**) AC-13; (**c**) SMA-13; (**d**) OGFC-13.

As can be seen in Figure 15, the grounding area of the tire becomes progressively smaller as the contact stress of the film increases. The 4LW film with the highest sensitivity was chosen to measure the tire contact area above 0.05 MPa, and this value was used as the effective tire–road contact area. The contact imprints of tires with different road surfaces are shown in Figure 16.

By analyzing the data presented in Table 7, several observations can be made. In the case of pavement without any texture (such as a steel plate) or with a slight texture depth, the tread rubber deformation can fully conform to the raised texture. As a result, the effective grounding area of the tire on the road surface is maximized. However, as the height of the raised texture on the road surface increases, the deformation of the tread rubber can only partially accommodate the raised texture. This leads to a reduction in the effective contact area between the tire and the road surface. Even with the minimum texture depth in the AC-13 pavement, the tread rubber can only partially conform to the road texture, resulting in a point contact state where the tire and asphalt pavement mainly make contact at the texture peaks. We calculated the tire contact reduction ratio, which represents the ratio of the effective contact area in the contour area of the imprints. On a flat steel plate, the tire imprint reduction rate is the highest. However, on actual asphalt pavements, the block pattern tire contact reduction rate decreases by approximately 5–10%, while the reduction rate for longitudinal pattern tires decreases by about 10–15%. The OGFC-13 pavement exhibits the lowest tire reduction rate. Furthermore, as the texture depth of the pavement increases, the contact between the tire and the pavement texture gradually decreases.

### 5.4. Analysis of Tire–Road Contact Stress Concentration Effect

**Figure 17:**
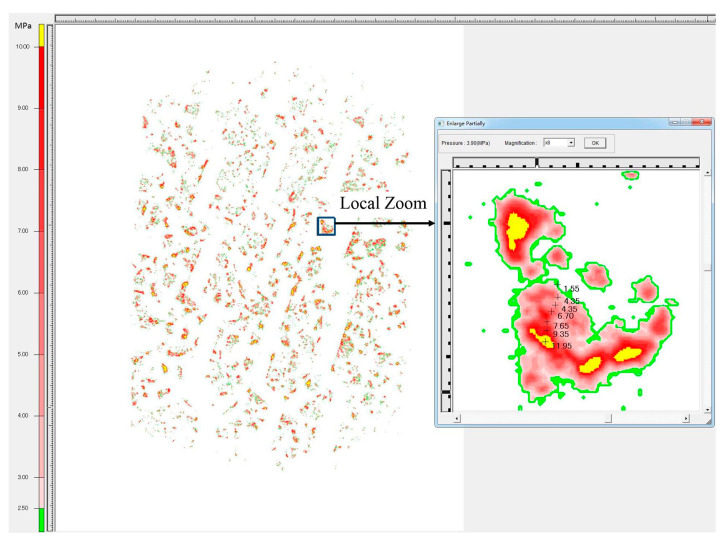
Contact stress concentration phenomenon of tires–roads.

When tires come into contact with rough surfaces, they typically experience point contact rather than full contact. This means that the actual contact area between the tire and the rough surface is significantly smaller than the nominal contact area of the surface. The size of the actual contact area has important implications for various properties, including thermal effects on contact surfaces, elastic deformation, viscous adhesion, and wear. These phenomena are closely linked to the stress concentration that occurs at the contact interface. The overall deformation of the tire occurs in order to balance the vehicle loads, but the tire tread rubber engages with the protruding textures on the road surface. The phenomenon of contact stress concentration at the top of pavement structure is obvious, as shown in Figure 17.

**Figure 18:**
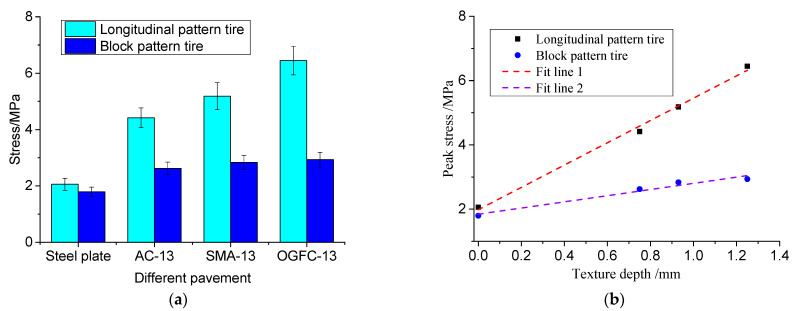
Peak contact stress and correlation with texture depth: (**a**) peak contact stress; (**b**) peak stress versus tectonic depth.

The pressure-sensitive film used in this study is flexible and deformable. When the rough texture of the road surface penetrates the tire rubber, the film undergoes folding deformation. In cases where the local texture is particularly sharp, the folding deformation of the film can be significant. This irregular folding can lead to the extrusion of the micro capsule materials, resulting in local stress concentration anomalies. To minimize system measurement errors in the film system, a statistical approach inspired by the extreme value distribution model in meteorology research was employed. Specifically, the tire contact stress value corresponding to the 95th quartile was calculated. This value serves to characterize the peak contact stress at the top of the pavement texture, as illustrated in Figure 18.

Figure 18 reveals significant differences in peak contact stresses among different road surfaces. The unconstructed steel plate exhibits the lowest peak contact stresses, primarily due to stress concentrations at the edges of the tread blocks. However, even on the flat steel plate, the tire peak contact stress surpasses the simplified uniform load specified in design standards. The rough asphalt pavement texture and tire engagement result in significantly higher tire peak contact stresses compared with a flat steel surface. The peak contact stress for car tires reaches around 3 MPa, while truck tires exhibit slightly lower values of approximately 2 MPa on flat steel plates. This difference can be attributed to variations in tire structure, tread rubber hardness, and tread block design. On rough asphalt pavement, the increase in ground peak stress for truck tires surpasses that of car tires, reaching 6 MPa or more. Hence, the contact stress of tires on actual road surfaces exhibits a significant non-uniform distribution, with peak stresses at texture peaks exceeding expectations. The correlation between peak contact stress and pavement texture depth for different tires is illustrated in Figure 18b. According to the calculation results in Table 8, a strong linear relationship between peak contact stress and pavement texture depth with correlation coefficients close to one is observed, indicating that greater pavement texture depth leads to a linear increase in texture peak stress. Rich pavement texture facilitates enhanced embedding and extrusion between the tread rubber and pavement, thereby improving the friction coefficient between the tire and pavement. These findings align with the conclusions drawn in the study by Yu et al. [43].

## 6. Findings and Future Study

(1) The pressure film test system utilizes pressure-sensitive microencapsulation materials and color-developing materials to display different color densities at different force which can be converted to pressure values. Thus, the contact pressure test between the tire and the road surface can be accurately tested. The relative error of the test is within 4%, and the contact stress at the top of the small texture can be identified.
(2) Under the rated tire pressure and load conditions, the outer contour of the tire’s grounding imprint exhibits an approximately rectangular shape. However, as the load decreases or the tire pressure increases, the tire imprint tends to become elliptical or even circular. Conversely, when the load increases or the tire pressure decreases, the imprint retains its width while the length increases, maintaining an approximately rectangular shape. The tire grounding area demonstrates a nearly linear relationship with both tire pressure and load, and changes in load have a more significant influence on the contact area compared with tire pressure.
(3) On level pavement, the tested light trucks exhibit a longitudinal tread reduction rate of 75–80%, while the block tread reduction rate was 65–70%. However, on actual asphalt pavement, the block tread tire contact reduction rate decreased by approximately 5–10%, and the longitudinal tread tire contact reduction rate decreased by approximately 10–15%. Additionally, as the texture depth of the pavement increased, the contact area of the tires with the pavement texture decreased.
(4) The embedding and meshing of the tire and the road surface result in significant stress concentration at the actual contact interface. Even on a flat steel plate surface, the peak stress at the edge of the tread block is much higher than the 0.7 MPa design uniform load, by approximately 2.5 to 3 times. On the asphalt pavement, the peak stress reaches 4 to 10 times the design uniform load, and this value increases as the texture depth of the pavement increases.

The pressure film test method mainly tests the static load, cannot study the influence of speed factors, and the test material cannot be reused. The meshing effect between the pavement rough texture and the tread rubber contributes to the stability of tire–road contact and tire friction. However, excessively high tire contact stress can negatively impact the wear life of the pavement texture and accelerate tire rubber abrasion. This is a multi-objective optimization topic. Therefore, it is essential to consider this issue comprehensively, and further in-depth research is needed to address it.

## Figures and Tables

**Table 1:** Prescale film types.

Product	Pressure Range/MPa	Product Size/W (mm) × L (mm)	Type
Super high pressure (HHS)	130~300	270 × 12	Mono-sheet type
High pressure (HS)	50~130	270 × 12	Mono-sheet type
Medium pressure (MS)	10~50	270 × 12	Mono-sheet type
Medium pressure (MW)	10~50	270 × 12	Two-sheet type
Low pressure (LW)	2.5~10	270 × 12	Two-sheet type
Super low pressure (LLW)	0.5~2.5	270 × 6	Two-sheet type
Ultra super low pressure (LLLW)	0.2~0.6	270 × 5	Two-sheet type
Extreme low pressure (4LW)	0.05~0.2	310 × 3	Two-sheet type

**Table 2:** Test results of different modified asphalt.

Test Items	Unit (of Measure)	SBS-Modified Asphalt	High Viscosity Modified Asphalt
Penetration (25 °C, 100 g, 5 s)	0.1 mm	54	36
Ductility (5 °C, 5 cm/min)	cm	33	25
Softening point T_R&B_	°C	84	96.0
Flash point	°C	338	326
Solubility	%	99.6	99.7
Storage stability (48 h softening point difference)	°C	1.4	2.3
Elastic recovery (25 °C)	%	98.3	99.2
Kinematic viscosity (135 °C)	Pa·s	2.94	--
Dynamic viscosity (60 °C)	Pa·s	8627	463,824

**Table 3:** Test results for evaluation of coarse aggregate properties.

Test Items	Unit (of Measure)	Test Results	Technical Requirement
Crushed stone value	%	13.8	≤20
Los Angeles Abrasion Losses	%	16.2	≤28
Apparent specific gravity	-	2.860	≥2.60
Water absorption	%	0.56	≤2.0
Adhesion with modified bitumen	classifier: step, level	5	5
Needle and plate particle content (>9.5 mm)	%	5.8	≤12
Water washing method <0.075 mm particles	%	0.4	≤1
Polished stone value	BPN	43	≥42

Note: “-” indicates that the item has no unit of measurement.

**Table 4:** Fine aggregate evaluation test results.

Test Items	Unit (of Measure)	Test Results	Technical Requirement
Apparent specific gravity	-	2.764	≥2.50
Soundness	%	3.6	≤12
Angularity	s	41	≥35
Sand equivalent	%	68	≥65

Note: “-” indicates that the item has no unit of measurement.

**Table 5:** Asphalt mixture gradation.

Gradation Type	Percentage Passage of Sieve Holes (mm)/%										
	19	16	13.2	9.5	4.75	2.36	1.18	0.6	0.3	0.15	0.075
AC-13	-	100	99.2	75.4	39.3	30.2	23.2	16.9	9.4	6.3	4.2
SMA-13	-	100	95.4	59.1	26.5	21.7	18.4	16.6	14.2	12.4	10
OGFC-13	-	100	94.9	69.6	20.2	12.8	10.7	8.2	6.1	5.6	4.1

**Table 6:** Linear regression coefficients of contact area with tire pressure and load.

Test Condition	*a*	*b*	*R* ^2^
Different load	906.56	7842.61	0.998
Different tire pressure	−11.85	27,596.00	0.987

**Table 7:** Tire contact area statistics for different road surfaces.

Pavement	Texture Depth/mm	Tires	Effective Contact Area/mm^2^	Reduction Rate/%	Tires	Effective Contact Area/mm^2^	Reduction Rate/%
plate	0	Longitudinal tread tire (670 kPa, 12.15 kN)	19,265	76.72	Block tread tires (400 kPa, 9.57 kN)	17,107	69.08
AC-13	0.75		18,933	65.78		16,437	65.24
SMA-13	0.93		18,563	62.03		14,929	60.92
OGFC-13	1.25		18,034	61.04		15,746	61.41

**Table 8:** Linear regression coefficients of different tire peak stresses and texture depths.

Test Conditions	*a*	*b*	*R* ^2^
Block tread tire (400 kPa, 9.57 kN)	0.961	1.844	0.949
Longitudinal tread tire (670 kPa, 12.15 kN)	3.471	1.984	0.993

## Data Availability

The data presented in this study are available on request from the corresponding author.
